# The effect of body mass index and physical activity on hypertension among Chinese middle-aged and older population

**DOI:** 10.1038/s41598-017-11037-y

**Published:** 2017-08-31

**Authors:** Wenzhen Li, Dongming Wang, Chunmei Wu, Oumin Shi, Yanfeng Zhou, Zuxun Lu

**Affiliations:** 1 Department of Social Medicine and Health Management, School of Public Health, Tongji Medical College, Huazhong University of Science and Technology, Wuhan, China; 2 Department of Occupational & Environmental Health, School of Public Health, Tongji Medical College, Huazhong University of Science and Technology, Wuhan, China; 3 Wuhan Hospital for the Prevention and Treatment of Occupational Diseases, Wuhan, China; 4 Hongqiao International Institute of Medicine, Shanghai Tongren Hospital/Faculty of Public Health, Shanghai Jiao Tong University School of Medicine, Shanghai, China

## Abstract

Few studies have been conducted to explore the independent and combined associations of body mass index (BMI) and physical activity with risk of hypertension in Chinese population. A cross-sectional study of 5291 individuals (aged ≥ 40 years) selected using multi-stage sampling method was conducted from October 2013 to December 2015. In the present analysis, 55.64% of the participants were women, and the mean age of participants was 55.37 ± 10.56. Compared with individuals in normal group, the risks of hypertension were nearly double in overweight subjects (odds ratio [OR] 1.77, 95% confidence interval [CI] 1.53–2.05) and more than three times higher in obese subjects (3.23, 2.62–4.13). Multi-adjusted odds for hypertension associated with low, moderate, and high physical activity were 1.44 (1.17–1.86), 1.40 (1.09–1.79) and 1.000, respectively. In comparison with normal weight subjects who reported high levels of physical activity, subjects who reported both low levels of physical activity and obesity showed the highest risk of hypertension (5.89, 3.90–8.88). In conclusion, both elevated BMI and reduced physical activity appear to play an important role in the risk of hypertension among Chinese middle-aged and older population. The risk of hypertension associated with overweight and obesity can be reduced considerably by increased physical activity levels.

## Introduction

Hypertension is one of the leading causes of global burden of disease and is widely acknowledged as the most common cardiovascular disorder and may account for a large proportion of stroke, coronary heart disease, renal failure and premature mortality^1^, affecting one in four adults in United States^2^ and worldwide^3^. The prevalence of hypertension is increasing globally, and it is estimated that hypertension is likely to rise to 30% by the year 2025^3^. In China, two nationwide blood pressure surveys suggested that 66% increase in the prevalence of hypertension from 1991 to 2002 with increasing prevalence of obesity and a sedentary lifestyle. The surveys also showed half of the elder people (60 years old and above) had hypertension^4,5^.

Despite of its high prevalence, hypertension has a low rate of successful treatment. In the United States for example, approximately two-thirds of adults with hypertension are either untreated or undertreated^6^. It may due to the known difficulty of successfully treating hypertension as well as its large health burden, more and better primary prevention through lifestyle modifications could become a major public health priority.

Previous studies have suggested that high body mass index (BMI)^7–9^, and low physical activity^10–12^ are modifiable risk factors for hypertension. However, other studies reported inconsistent results. A prospective study^13^ reported that Harvard college alumni who did not participate in vigorous exercise had a 35% higher incidence of hypertension than those who were more active. Haapenan *et al*.^14^ found the intensity of activity was not associated with the risk of hypertension after adjustment for both age and total amount of activity. The Atherosclerosis Risk in Community Study (ARIC) demonstrated the inverse association between leisure time physical activity and risk of hypertension in middle-aged white men, but lack of association in white women^15^. A Japanese prospective study^16^ showed that the duration of walking to work and other types of physical activity were associated with a reduction in the risk for hypertension in Japanese men. Moreover, few studies were conducted to explore their combined effects on hypertension, especially in Chinese population.

We conducted a cross-sectional study to examine the individual and combined effects between BMI and physical activity in association with hypertension in Chinese middle-aged and older population, which may be an important supplementary on studies of hypertension and help inform more effective interventions.

## Results

Among the 5,291 participants, 55.64% of the participants were women, and the mean age of participants was 55.37 ± 10.56, 31.90% of participants were hypertensive. Characteristics of the participants by categories of BMI and physical activity were shown in Table 1. Overall, 2,724 (51.48%) individuals reported BMI were normal (18.5–23.9), and 477 (9.02%) ≥ 28, respectively. There were 3,426 (64.75%) subjects reporting low physical activity, and only 476 (9.00%) reporting high physical activity. There were significant differences between different categories of BMI in most of the covariates and the significant differences between different categories of physical activity were found in the covariates of marital status, educational level, smoking status and diabetes status.

**Table 1:** Characteristics of study population by BMI and physical activity category.

Characteristics	BMI n (%)				*P*	Physical Activity n (%)			*P*
	Underweight	Normal	Overweight	Obese		low	moderate	high	
	214 (4.04)	2724 (51.48)	1876 (35.47)	477 (9.02)		3426 (64.75)	1389 (26.25)	476 (9.00)	
**age, mean years (±SD)**	61.59 (12.76)	55.16 (10.89)	55.33 (9.78)	53.95 (9.49)	<0.001	55.37 (10.75)	55.37 (10.24)	55.37 (10.09)	0.998
Sex					0.174				0.212
male	82 (38.32)	1209 (44.38)	854 (45.52)	202 (42.35)		1522 (44.42)	598 (43.05)	227 (47.69)	
female	132 (61.68)	1515 (55.62)	1022 (54.48)	275 (57.65)		1904 (55.58)	791 (56.95)	249 (53.31)	
**Waist circumference**					<0.001				0.855
normal	205 (95.9)	2138 (78.49)	768 (40.94)	36 (7.55)		2047 (59.75)	818 (58.89)	282 (59.24)	
abnormal	9 (4.21)	586 (21.51)	1108 (59.06)	441 (92.45)		1379 (40.25)	571 (41.11)	194 (40.76)	
**Marital status**					<0.001				0.024
single	2 (0.93)	36 (1.32)	20 (1.07)	6 (1.26)		53 (1.55)	9 (0.65)	2 (0.42)	
married	177 (82.71)	2515 (92.33)	1774 (94.56)	454 (95.18)		3173 (92.62)	1294 (93.16)	453 (95.17)	
divorced	2 (0.93)	38 (1.40)	13 (0.69)	6 (1.26)		31 (0.90)	23 (1.66)	5 (1.05)	
widowed	33 (15.42)	126 (4.63)	65 (3.46)	11 (2.31)		160 (4.67)	59 (4.25)	16 (3.36)	
other	0	9 (0.33)	4 (0.21)	0		9 (0.26)	4 (0.29)	0	
**Educational level**					<0.001				<0.001
Primary school and below	92 (42.99)	631 (23.16)	314 (16.74)	62 (13.00)		827 (24.14)	200 (14.40)	72 (15.13)	
Junior middle school	67 (31.31)	955 (35.06)	730 (38.91)	196 (41.09)		1251 (36.51)	526 (37.87)	171 (35.92)	
Senior middle school	36 (16.82)	719 (26.40)	534 (28.46)	139 (29.14)		869 (25.36)	416 (29.95)	143 (30.04)	
College degree and above	19 (8.88)	419 (15.38)	298 (15.88)	80 (16.77)		479 (13.98)	247 (17.78)	90 (18.91)	
**Smoking status**					0.001				0.016
no	164 (76.64)	2166 (79.52)	1563 (83.32)	401 (84.07)		2746 (80.15)	1143 (82.29)	405 (85.08)	
yes	50 (23.36)	558 (20.48)	313 (16.68)	76 (15.93)		680 (19.85)	246 (17.71)	71 (14.92)	
**Drinking status**					0.281				0.451
no	185 (86.45)	2232 (81.94)	1527 (81.40)	397 (83.23)		2827 (82.52)	1125 (80.99)	389 (81.72)	
yes	29 (13.55)	492 (18.06)	349 (18.60)	80 (16.77)		599 (17.48)	264 (19.01)	87 (18.28)	
**Diabetes mellitus**					<0.001				0.004
no	193 (90.19)	2437 (89.46)	1651 (88.01)	420 (88.05)		3044 (88.85)	1253 (90.21)	404 (84.47)	
yes	6 (2.80)	148 (5.43)	165 (8.80)	48 (10.06)		225 (6.57)	96 (6.91)	46 (9.66)	
unknown	15 (7.01)	139 (5.10)	60 (3.20)	9 (1.89)		157 (4.58)	40 (2.88)	26 (5.46)	

BMI, body mass index; SD, standard deviation.

Stratification analyses showed that the association between larger BMI and hypertension risk was noticeable in both of those abnormal waist circumferences and without DM (Table 2). And this association was stronger in males and those with college and above educational level, those ages < 60 years old, married, nondrinkers and nonsmokers (Table 2). In terms of lower physical activity, the association with hypertension risk was inconsistent and additionally significant among female and individuals with senior middle school educational level and college degree and above. In addition, the association between physical activity and hypertension risk was also found among those without DM, those aged < 60 years old, married, nondrinkers and nonsmokers. As to waist circumference, abnormal waist with low physical activity got the highest risk (adjusted OR 1.60, 95% CI 1.14–2.24) (Table 2).

**Table 2:** Adjusted odd ratios (95%CI) for hypertension risk according to BMI and physical activity stratified by major characteristics.

Characteristics (n)	BMI				Physical Activity		
	Underweight	Normal	Overweight	Obese	low	moderate	high
**Age**							
<60 (3448)	0.53 (0.25,1.10)	Ref.	2.05 (1.69,2.48)	3.56 (2.68,4.73)	1.60 (1.16,2.20)	1.60 (1.14,2.26)	Ref.
≥60 (1843)	0.52 (0.33,0.82)	Ref.	1.41 (1.12,1.77)	2.62 (1.70,4.03)	1.34 (0.96,1.87)	1.19 (0.83,1.71)	Ref.
**Sex**							
male (2347)	0.56 (0.31,1.03)	Ref.	1.83 (1.47,2.27)	3.64 (2.51,5.28)	1.37 (0.99,1.90)	1.23 (0.86,1.75)	Ref.
female (2944)	0.54 (0.33,0.89)	Ref.	1.68 (1.38,2.05)	2.89 (2.13,3.92)	1.63 (1.18,2.25)	1.63 (1.16.2.30)	Ref.
**Waist circumference**							
normal (3147)	0.48 (0.33,0.75)	Ref.	1.86 (1.54,2.25)	2.40 (1.17,4.91)	1.39 (1.02,1.90)	1.45 (1.04,2.03)	Ref.
abnormal (2144)	1.51 (0.37,6.24)	Ref.	1.64 (1.31,2.06)	3.04 (2.30,4.01)	1.60 (1.14,2.24)	1.39 (0.97,1.99)	Ref.
**Marital status**							
Single (64)	—	Ref.	0.72 (0.09,5.77)	0.56 (0.02,20.85)	0.15 (0.01,6.04)	1.09 (0.02,57.41)	Ref.
Married (4920)	0.57 (0.37,0.88)	Ref.	1.78 (1.53,2.07)	3.21 (2.52,4.09)	1.59 (1.25,2.01)	1.49 (1.16,1.92)	Ref.
Divorced (59)	—	Ref.	—	—	—	0.20 (0.01,9.75)	Ref.
Widowed (235)	0.49 (0.18,1.28)	Ref.	1.27 (0.61,2.61)	1.31 (0.27,6.35)	0.68 (0.22,2.09)	0.89 (0.27,2.96)	Ref.
Other (13)	—	Ref.	—	—	—	—	Ref.
**Educational level**							
Primary school and below (1099)	0.67 (0.37,1.21)	Ref.	1.37 (0.98,1.93)	1.29 (1.04,3.59)	0.95 (0.54,1.67)	1.30 (0.71,2.41)	Ref.
Junior middle school (1948)	0.61 (0.32,1.19)	Ref.	1.79 (1.41,2.28)	2.89 (1.99,4.22)	1.35 (0.93,1.96)	1.54 (1.04,2.29)	Ref.
Senior middle school (1428)	0.32 (0.11,0.96)	Ref.	2.00 (1.52,2.64)	3.87 (2.48,6.04)	1.87 (1.21,2.88)	1.41 (0.89,2.25)	Ref.
College degree and above (816)	0.29 (0.06,1.37)	Ref.	1.59 (1.10,2.31)	4.46 (2.44,8.16)	2.00 (1.12,3.57)	1.35 (0.73,2.51)	Ref.
**Smoking status**		Ref.					Ref.
no (4294)	0.48 (0.31,0.75)	Ref.	1.75 (1.50,2.05)	3.21 (2.49,4.15)	1.63 (1.27,2.10)	1.46 (1.12,1.91)	Ref.
yes (997)	0.82 (0.39,1.75)	Ref.	1.77 (1.25,2.51)	2.97 (1.63,5.41)	0.88 (0.50,1.56)	1.21 (0.66,2.21)	Ref.
**Drinking status**							
no (4341)	0.51 (0.34,0.77)	Ref.	1.78 (1.52,2.09)	3.54 (2.73,4.58)	1.54 (1.19,1.99)	1.50 (1.14,1.98)	Ref.
yes (950)	0.92 (0.33,2.56)	Ref.	1.55 (1.09,2.20)	1.72 (0.95,3.11)	1.28 (0.76,2.17)	1.11 (0.63,1.94)	Ref.
**Diabetes mellitus**							
no (4701)	0.55 (0.36,0.83)	Ref.	1.76 (1.51,2.06)	3.29 (2.57,4.22)	1.49 (1.16,1.91)	1.44 (1.10,1.88)	Ref.
yes (367)	0.35 (0.06,0.17)	Ref.	1.41 (0.84,2.37)	2.14 (0.95,4.81)	1.46 (0.74,2.89)	1.38 (0.65,2.92)	Ref.
unknown (223)	0.69 (0.16,2.98)	Ref.	1.50 (0.61,3.66)	2.18 (0.41,11.53)	1.51 (0.37,6.20)	1.23 (0.24,6.12)	Ref.

The multivariate model adjusted for age, sex, BMI (<18.5, 18.5~23.9, 24~28, ≥28), physical activity (0~600met-hours/week; 600~1200 met-hours/week; ≥1200 met-hours/week); waist circumference (male, ≤90, >90; female, ≤80, >80), marital status, education level, smoking status, drinking status and diabetes mellitus, except for the stratified variable itself.

BMI, body mass index.

**Figure 1:**
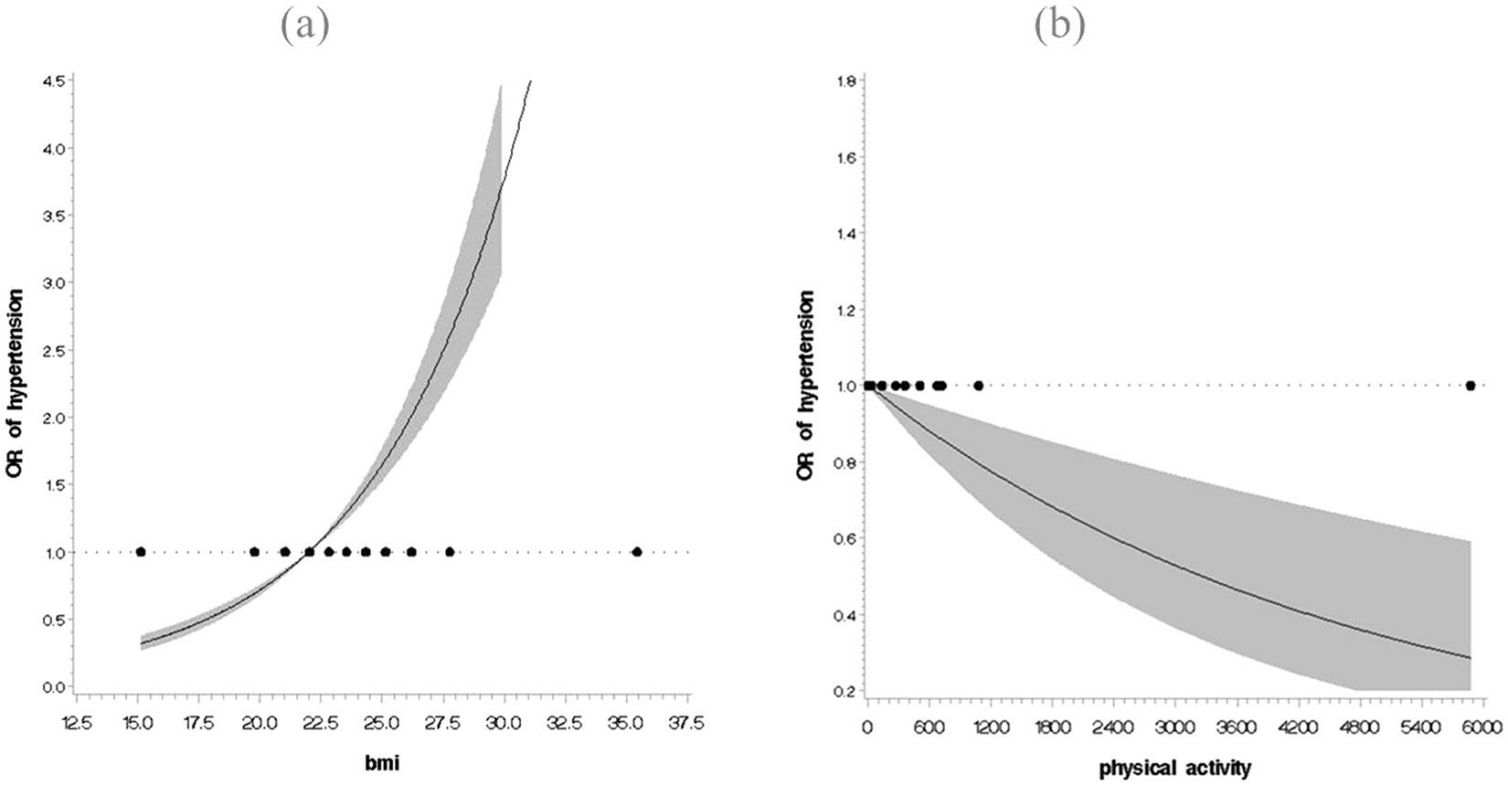
Multivariable adjusted spline curves for relation between BMI, physical activity and risk of hypertension. (**a**) BMI and risk of hypertension, (**b**) physical activity and risk of hypertension.

After multivariate adjustment for potential confounders, we found that higher BMI and lower level of physical activity were independently associated with a higher risk of hypertension. Table 3, Fig. 1(a) and (b) showed that the individual effects of BMI and physical activity on hypertension risk. Compared with normal weight subjects, the odds of hypertension were nearly double (adjusted OR 1.77, 95% CI 1.53–2.05) in overweight subjects and more than three times higher (adjusted OR 3.23, 95% CI 2.62–4.13) in obese subjects, however, a protective effect was presented in underweight subjects (adjusted OR 0.47, 95% CI 0.32–0.69). Multi-adjusted odds for hypertension associated with low, moderate, and high level of physical activity were 1.44 (1.17–1.86), 1.40 (1.09–1.79) and 1.00, respectively. The spline curve in Fig. 1(a) and (b) illustrated that there were nonlinear associations of BMI and physical activity with hypertension risk (*P* < 0.001). Cubic spline regression confirmed the risk of hypertension may be higher with higher BMI and lower level of physical activity.

**Table 3:** Odds ratios for association between each of BMI and physical activity and risk of hypertension.

	OR (95%CI)^1^	Adj-OR (95%CI)^2^	Adj-OR (95%CI)^3^
**BMI**			
underweight	0.62 (0.43–0.90)	0.48 (0.32–0.71)	0.47 (0.32–0.69)
normal	1.00	1.00	1.00
overweight	1.94 (1.71–2.21)	1.77 (1.53–2.05)	1.77 (1.53–2.05)
obese	3.49 (2.86–4.26)	3.54 (2.65–4.25)	3.23 (2.62–4.13)
**MET**			
Low	1.26 (1.02–1.57)	1.49 (1.18–1.87)	1.44 (1.17–1.86)
Moderate	1.30 (1.03–1.63)	1.40 (1.10–1.80)	1.40 (1.09–1.79)
High	1.00	1.00	1.00

^1^Unadjusted.

^2^Adjusted for age, sex, educational level, marital status, smoking status, drinking status, waist and diabetes mellitus.

^3^Adjusted for age, sex, educational level, marital status, smoking status, drinking status, waist, diabetes mellitus and BMI (or physical activity).

BMI, body mass index; MET, metabolic equivalent.

The joint associations of different levels of BMI and physical activity with the risk of hypertension were showed in Table 4. The odds of hypertension were 54% higher in normal weight inactive subjects than in normal weight high active subjects (adjusted OR 1.54, 95% CI 1.09–2.18). In comparison with normal weight subjects who reported high level of physical activity, subjects who reported both low level of physical activity and had obese weight showed the highest risk of hypertension (adjusted OR 5.89, 95% CI 3.90–8.88).

**Table 4:** Odds ratios for the joint effect of BMI and physical activity on risk of hypertension.

BMI	physical activity		
	low	moderate	high
**Unadjusted odds ratios (95% CI)**			
underweight	0.68 (0.40–1.15)	1.31 (0.60–2.85)	0.38 (0.05–3.03)
normal	1.18 (0.84–1.63)	1.47 (1.04–2.09)	1.00
overweight	2.58 (1.86–5.38)	2.18 (1.53–3.10)	2.01 (1.30–3.1)
obese	4.87 (3.35–7.08)	3.51 (2.15–5.73)	2.58 (1.25–5.33)
**Adjusted odds ratios (95% CI)** ^**a**^			
underweight	0.65 (0.37–1.13)	1.25 (0.54–2.89)	0.33 (0.04–2.83)
normal	1.54 (1.09–2.18)	1.70 (1.17–2.45)	1.00
overweight	2.91 (2.04–4.14)	2.45 (1.68–3.57)	2.22 (1.40–3.53)
obese	5.89 (3.90–8.88)	3.91 (2.30–6.64)	3.00 (1.39–6.50)

^a^Adjusted for age, sex, educational level, marital status, smoking status, drinking status, waist and diabetes mellitus.

BMI, body mass index.

## Discussion

In this cross-sectional study, we found that high BMI and low physical activity were independently associated with hypertension in middle-age and older population in China, after adjusting for socioeconomic factors and DM. Moreover, the combined effect of low physical activity and high BMI was associated with the highest risk of hypertension. Third, the study also indicated that high level of physical activity might be a protective factor of hypertension, and it could not offset the risk of obesity, which suggests that preventive interventions including weight control and physical activity are both very important, even among those with normal BMI.

To our knowledge, this study is the first to examine the independent and combined effects of physical activity and BMI on the risk of hypertension simultaneously among Chinese adults. We found that the risk of hypertension accelerated with the increase of BMI, which was similar with other studies^7,17–19^. And it also confirmed that overweight and obesity were risk factors for health, just as we all know. As to the physical activity, our results might be different from some studies in western countries, which reported physical activity was not associated with hypertension. It may be mainly owing to the population with different areas and ethnicity. Besides, metabolic equivalent was calculated using different methods for each activity may also contribute to it.

In addition to indirect effects by reducing body fat, the decrease in risk of hypertension induced by physical activity may be explained through attenuation of adrenergic sympathetic activity^20,21^ accompanied by the decreased systemic vascular resistance^22^, and decreased stroke volume combined with decreased contractility of the heart after exercise^23^. Other possible mechanisms include reduced levels of catecholamines^24^, as well as increased urinary sodium excretion and reduction of total peripheral resistance^25^.

As far as we know, only two studies examined the combined effect of BMI and physical activity on hypertension^8,9^, and the results were inconsistent. Gang Hu’ study^9^ in Finland showed higher BMI was not associated with hypertension (OR, 1.13, 95%CI 0.96–1.33 in men; OR, 1.13, 95%CI 0.96–1.33 in women). In contrast, our study showed a 77% increase in the risk of hypertension among overweight subjects. The differences between the two studies may be explained in part by the diversity of physical activity measurements and BMI category standard. Besides, BMI was categorized according to the guidelines of Working Group on Obesity in China in our study, however, the previous study according standardized protocol^26^. Similar to Caroline Jackson’ study^8^ conducted among Australia women, our findings also found that high levels of physical activity may reduce hypertension risk among overweight and obese population, but could not completely negate the increased risk of hypertension associated with overweight and obesity, which could provide new perspectives for hypertensive population.

There are some limitations in our study. First, the assessment of physical activity is self-reported and thus is imprecise. Misclassification is inevitable and may result in an under/over-estimation of the association of physical activity with the risk of hypertension. Second, the results of the present study are limited to middle-aged and older people and thus the findings cannot necessarily be extrapolated to younger people or other ethnicities. Further study is therefore warranted in more diverse populations. Third, although a number of confounders were adjusted in our study, there were still some other factors which were not included such as consumption of diet the family history of hypertension, which was also reported to be associated with hypertension risk in other studies^27,28^. Finally, this is cross-sectional study and thus the causal association of BMI and physical activity and risk of hypertension should be further examined in large cohort studies.

## Conclusion

In summary, both elevated BMI and reduced physical activity appear to play an important role in the risk of hypertension. The highest risk of hypertension was in obese subjects, who reported low level of physical activity, and the risk of hypertension associated with overweight and obesity may be reduced considerably by increased physical activity levels. Besides, there is a higher risk of hypertension among low physical activity subjects with normal weight population.

## Methods

### Ethical Approval

Our study was approved by the Ethics Committee of Tongji Medical College Institutional review Board, Tongji Medical College, Huazhong University of Science and Technology (Wuhan, Hubei, China) and was carried out in accordance with the principles of the Declaration of Helsinki. All the participants were provided with the informed consent before participation in the study, and all questionnaires were filled in by participants anonymously.

### Study design and population

The survey was conducted in Zhuhai and Shenzhen city, two cities in Southeast China from October 2013 to December 2015. It was aimed to provide information for community diagnosis for local health sectors. Respondents were selected using multi-stage sampling method, and face-to-face interviews were conducted by trained local interviewers using a questionnaire to subjects with informed consent. The information of socio-economic characteristics, health status and health risk factors were collected and physical examination was also conducted among participants in community health service centers for blood pressure, height, weight and waist circumference. Eligible participants for the study were people over 40 years old in the survey.

### Ascertainment of hypertension

Blood pressure was measured via mercury sphygmomanometer and the participants were in sitting position. Each participant was examined three times of resting blood pressure in this study. All measurements and medical examinations were performed by trained nurses or physicians. Individuals were defined as hypertensive if they met one of the following standards: (1) self-reported physician diagnosis of hypertension; (2) self-reported current use of an antihypertensive medication; (3) mean value of systolic blood pressure (SBP) above 140 mmHg or/and diastolic blood pressure (DBP) above 90 mmHg measured in the medical examination.

### Assessment of BMI, Physical Activity and Covariates

BMI was computed as weight in kilograms divided by height in meters squared. During the analysis, the BMI category was categorized as underweight (<18.5 kg m^−2^), normal weight (18.5–23.9 kg m^−2^), overweight (24–27.9 kg m^−2^) and obesity (≥28 kg m^−2^) based on the guidelines of Working Group on Obesity in China^29^.

Frequency and average duration of each type of physical activity were obtained via the questionnaires: walking, biking, tai chi, qi gong, jogging, swimming, dancing, and playing basketball, badminton, or soccer. To estimate total energy expenditure (TEE) from physical activity, separate metabolic equivalent (MET) minutes per week were calculated for each activity according to the following formulas: MET coefficient of activity*duration (minutes per time)* frequency (times per week). Using the compendium of physical activities, METs per hour used for leisure activities were: 3 for walking, 4 for biking, 4.5 for tai chi and qi gong, 5.0 for dancing, 6.0 for doing exercise in gym, 7.0 for jogging, swimming and playing ball games^30^. The physical activity was divided into three groups according to the TEE: <600 MET- min/week, 600–1200 MET- min/week, and ≥1200 MET- min/week.

Waist circumference was recorded in centimeters and measured with the subject standing and wearing only underwear, using a nonstretching measuring tape positioned at the level midway between the lower edge of the last rib and the upper portion of the iliac crest. Waist circumference values of up to 90 cm in males and 80 cm in females were considered normal^31^. Other variables that may be associated with hypertension were obtained from the survey. The following were used as adjustment variables: age, gender, educational level, marital status, current smoking status, current drinking status, diabetes mellitus (DM) diagnosed by a physician were reported by the participants.

### Statistical Analysis

Sociodemographic characteristics of the participants were reported as mean (standard deviation, SD) for continuous variables and as number (percentages) for categorical variables. Logistic regressions were performed to evaluate the independent and combined associations of BMI and physical activity with hypertension. Covariates included age, gender, marital status, educational level, DM, smoking status, drinking status, physical activity, waist circumference, and BMI category. The results were presented as odd ratios (ORs) with 95% confidence intervals (CIs), with BMI of 18.5–23.9 and physical activity of ≥1200 MET-min/week as the reference groups. Nonlinear trends of hypertension risk were tested by restricted cubic spline logistic regression using 3 knots placed at the 10th, 50th, and 90th percentiles of BMI and physical activity, respectively, with 22 for BMI and 0 MET-min/weeks for physical activity as the reference groups. Stratified analyses were performed by baseline characteristics (including age (<60, ≥60 years), sex, waist circumference (normal, abnormal), marital status (single, married, divorce, widowed, other), educational level (primary school and below, junior middle school, senior middle school, college degree and above), current smoking (yes, no), current drinking (yes, no), DM (yes, no). Additionally, we tested the potential interaction by adding interaction terms of these covariates with BMI or physical activity, respectively. Moreover, we also estimated the combined effects of BMI and physical activity on the risk of hypertension, taking BMI 18.5–23.9 and physical activity 600–1200 MET-min/week as the reference category. All statistical analyses were performed using SAS version 9.3 software (SAS institute Inc., Cary, NC). A two-tailed *P*-value < 0.05 was considered statistically significant.

### Data Availability

The datasets generated during and/or analyzed during the current study are available from the corresponding author on reasonable request.
