# Author Correction: The effect of body mass index and physical activity on hypertension among Chinese middle-aged and older population

**DOI:** 10.1038/s41598-018-23080-4

**Published:** 2018-03-14

**Authors:** Wenzhen Li, Dongming Wang, Chunmei Wu, Oumin Shi, Yanfeng Zhou, Zuxun Lu

**Affiliations:** 1 Department of Social Medicine and Health Management, School of Public Health, Tongji Medical College, Huazhong University of Science and Technology, Wuhan, China; 2 Department of Occupational & Environmental Health, School of Public Health, Tongji Medical College, Huazhong University of Science and Technology, Wuhan, China; 3 Wuhan Hospital for the Prevention and Treatment of Occupational Diseases, Wuhan, China; 4 Hongqiao International Institute of Medicine, Shanghai Tongren Hospital/Faculty of Public Health, Shanghai Jiao Tong University School of Medicine, Shanghai, China

Correction to: *Scientific Reports* 10.1038/s41598-017-11037-y, published online 31 August 2017

This Article contains an error in the Acknowledgements section.

“Fundamental Research Funds for the Central Universities, Huazhong University of Science and Technology (2016YXMS215), Wuhan, China.”

should read:

“National Natural Science Foundation of China (NSFC, 71373090, “Study on the gatekeeper policy of CHS”).”

